# Supplementary figures and images for: Changes in environmental and engineered conditions alter the plasma membrane lipidome of fractured shale bacteria
Source: Microbiol Spectr. 2023 Dec 7;12(1):e02334-23. doi: 10.1128/spectrum.02334-23 (PMC10782966; doi:10.1128/spectrum.02334-23)

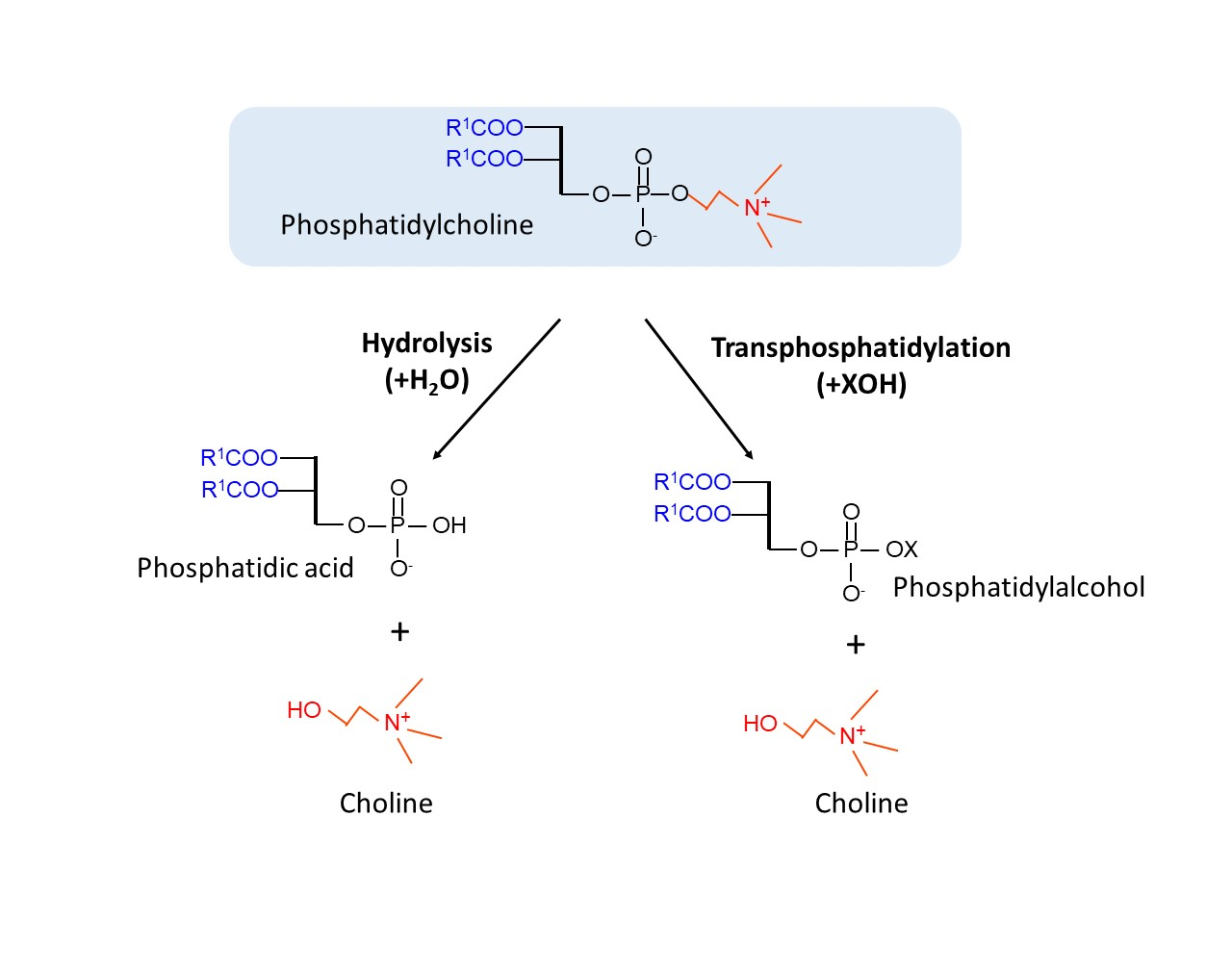

Supplement: Figure S1 — Phosphatidylcholine turnover. [file spectrum.02334-23-s0001.tiff]

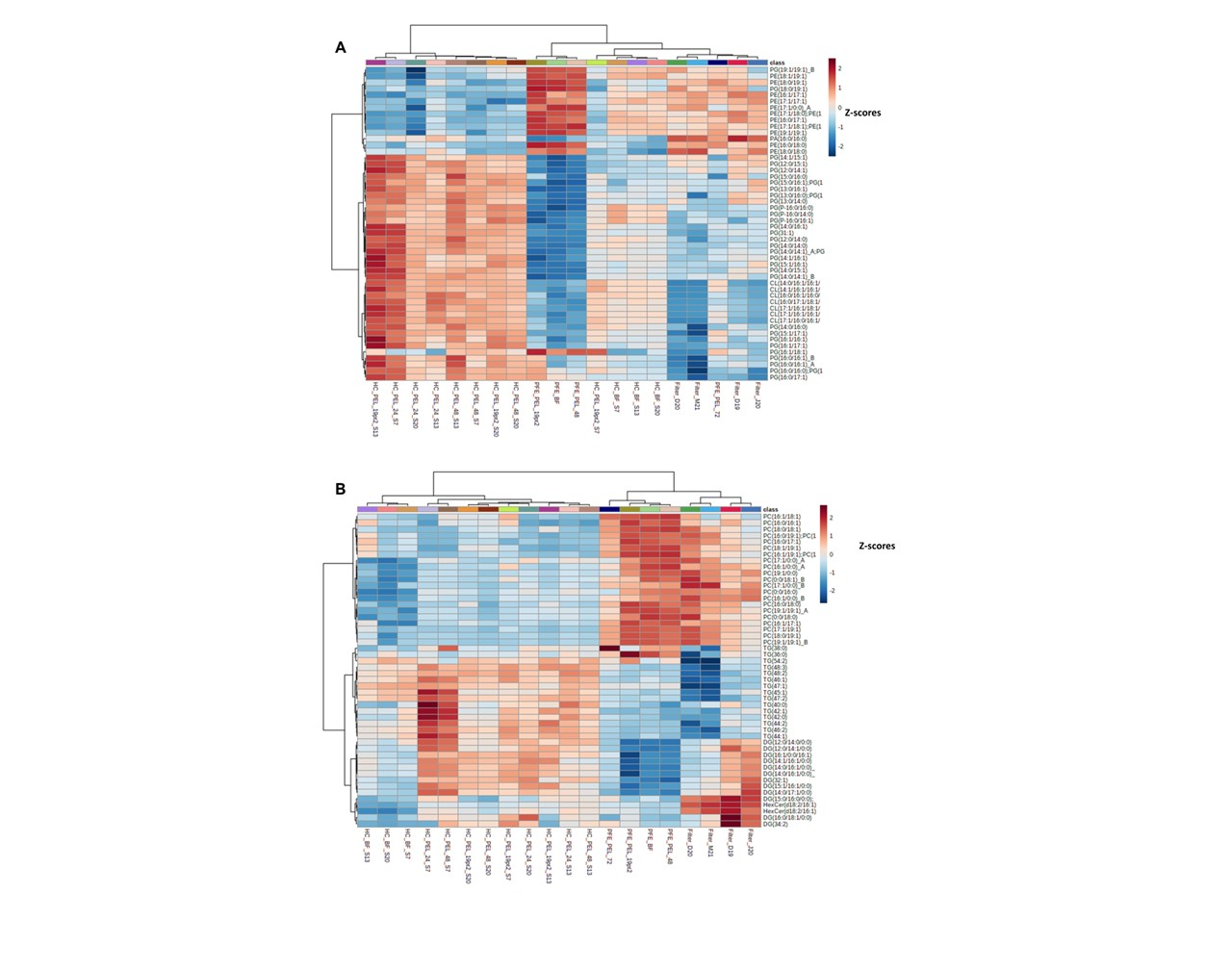

Supplement: Figure S2 — Heatmap of discriminant lipids. [file spectrum.02334-23-s0002.tiff]

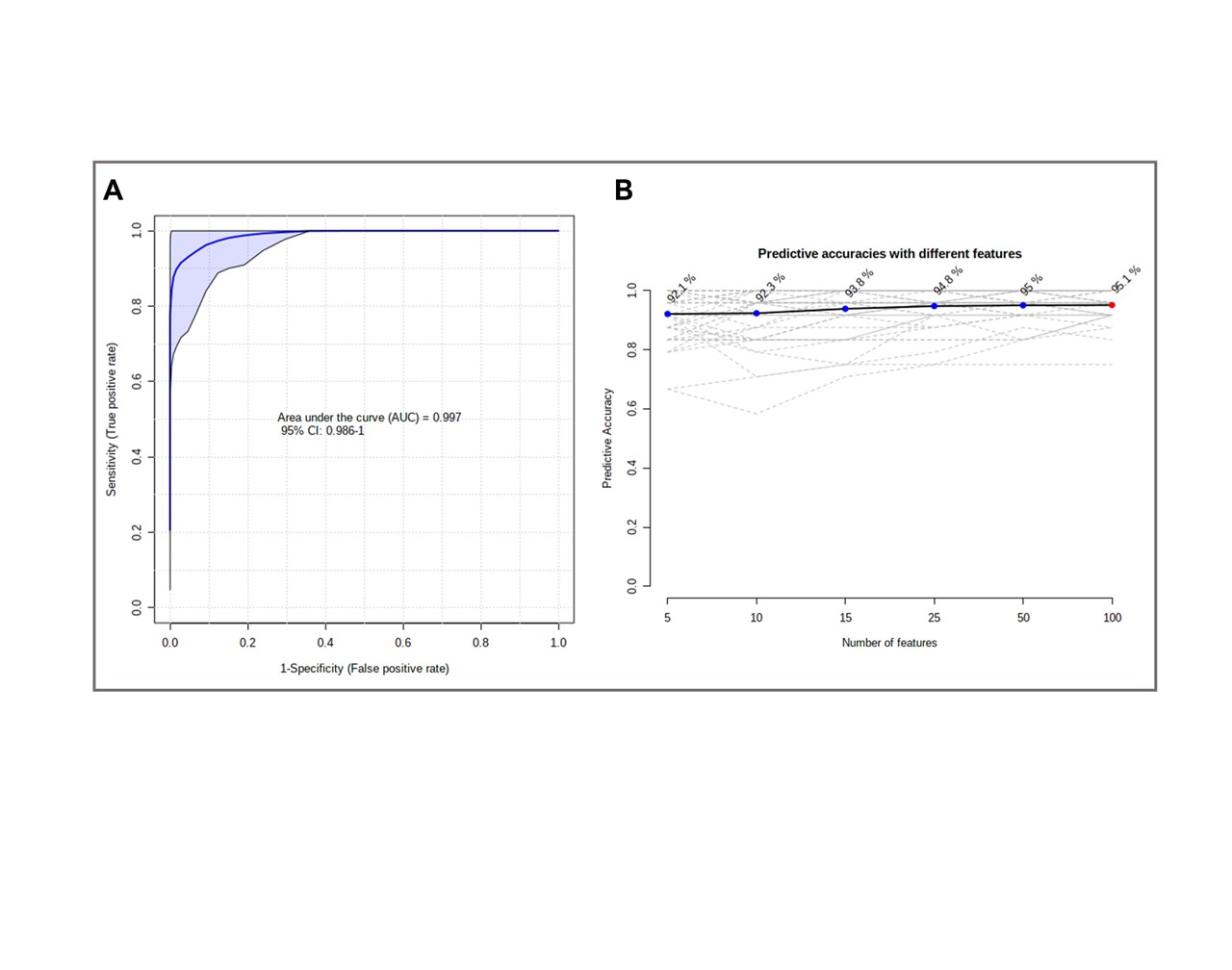

Supplement: Figure S3 — Validation of models. [file spectrum.02334-23-s0003.tiff]

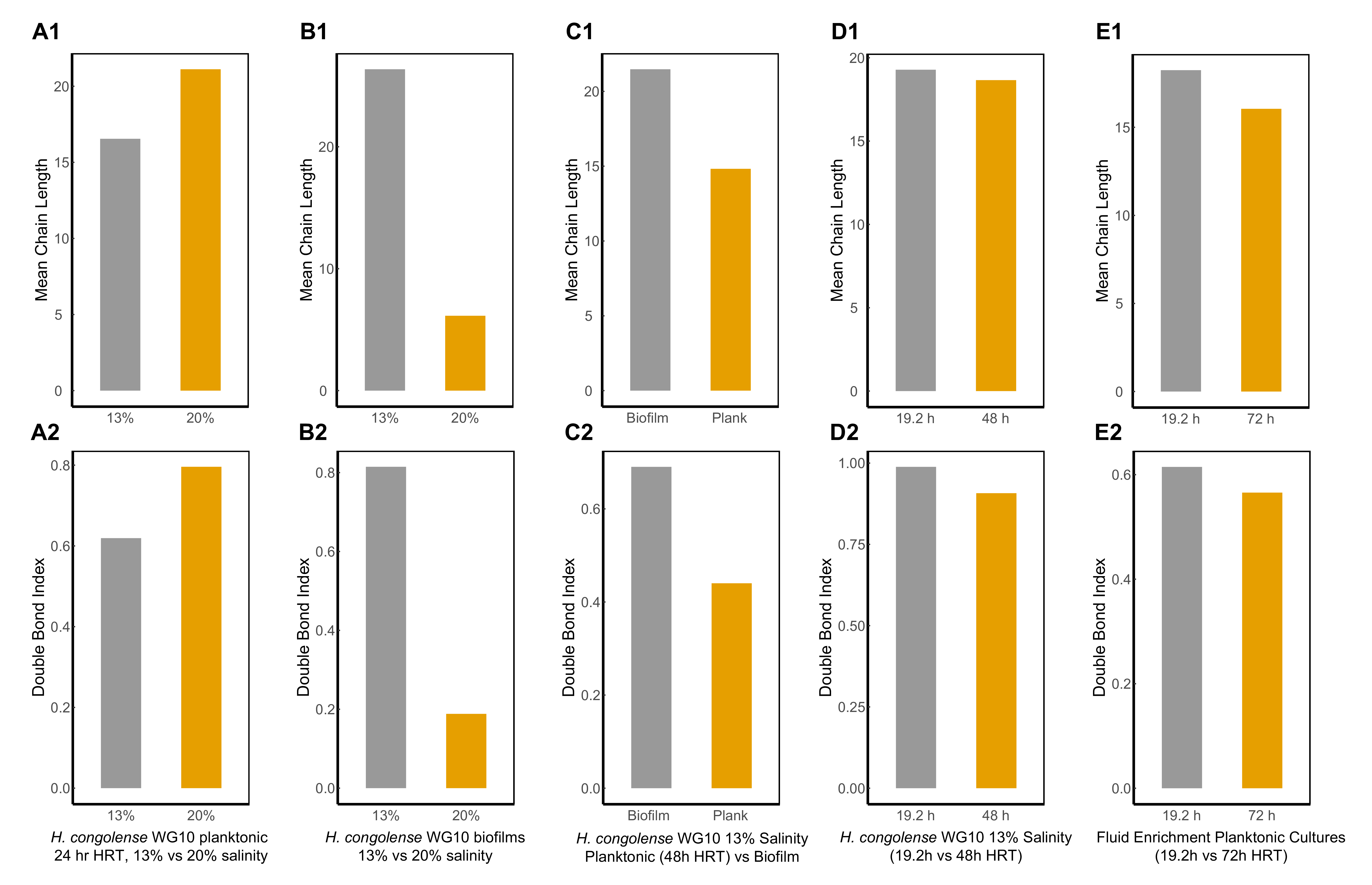

Supplement: Figure S4 — Double bond and chain length variations. [file spectrum.02334-23-s0004.tiff]
