# Supplementary material for: Changes in environmental and engineered conditions alter the plasma membrane lipidome of fractured shale bacteria
Source: Microbiol Spectr. 2023 Dec 7;12(1):e02334-23. doi: 10.1128/spectrum.02334-23 (PMC10782966; doi:10.1128/spectrum.02334-23)
Supplement: Supplementary Appendix Text — Supplementary text and tables. [file spectrum.02334-23-s0005.docx]

**Changes in environmental and engineered conditions alter the plasma membrane lipidome of fractured shale bacteria**

Chika Jude Ugwuodo,^a,b^ Fabrizio Colosimo,^c^ Jishnu Adhikari,^d^ Kent Bloodsworth,^e^ Stephanie A. Wright,^e^ Josie Eder,^e^ Paula J. Mouser^b^#

^a^Natural Resources and Earth Systems Science, University of New Hampshire, Durham, New Hampshire, USA

^b^Department of Civil and Environmental Engineering, University of New Hampshire, Durham, New Hampshire, USA

^c^New England Biolabs, Ipswich, Massachusetts, USA

^d^Sanborn, Head and Associates, Inc., Concord, New Hampshire, USA

^e^Biological Sciences Division, Pacific Northwest National Laboratory, Richland, Washington, USA

#Address correspondence to Paula J. Mouser, paula.mouser@unh.edu.

**SUPPLEMENTARY APPENDIX**

*Phosphatidylcholine recycling*

Most bacteria, fungi, plants and animals express functional phospholipase D (PLD; E.C.3.1.4.4) which catalyzes the hydrolysis and transphosphatidylation of phosphatidylcholine (PC) (1). In the first reaction, PC is broken down into phosphatidic acid and free choline. On the other hand, transphosphatidylation involves the introduction of an alcohol moiety to split PC into the corresponding phosphatidylalcohol (with modified polar headgroup) and free choline.

*High levels of Diacylglycerol*

Diacylglycerol is a neutral lipid comprising two acyl chains esterified to a glycerol moiety. It is not a common plasma membrane lipid in bacteria, which is why its relatively high abundance across incubation conditions in *H. congolense* WG10, was intriguing. We believe that DG accumulation in *H. congolense* WG10 primarily results from high phospholipid turnover during induced adjustments in membrane lipidome chemistry (2) and production of membrane-derived oligosaccharides (MDOs) in the periplasmic space. The latter is known to occur in *E. coli* under certain conditions of environmental stress (3,4) even though its role is still not very well understood. When required, DG is then converted by diacylglycerol kinases to phosphatidic acid, the immediate precursor of glycerophospholipids. From an efficiency standpoint, this is highly convenient as it offers a pathway for quicker response to the cell’s need for remodeling the membrane lipidome compared to *de novo* synthesis. Moreover, there is also a possibility that shale bacteria have evolved to accumulate DGs as a secondary energy source during nutrient depletion, which is very characteristic of engineered subsurface energy systems. This is even more plausible considering that some prokaryotes store energy in a similar neutral lipid, triacylglycerol (TG) (5). Additional studies are needed to confirm our observation of high DG accumulation and explore its potential roles in the persistence of shale taxa.

*Trends in fatty acid features of discriminant lipids*

An earlier study by our group (6) found that membrane fluidity (estimated as double bond index (DBI)) and thickness (estimated as mean (acyl) chain length (MCL)) of *H. congolense* WG10 planktonic cells increased under high salinity (20% NaCl) compared to the optimum (13% NaCl). In the present study, we reported that the MCL and DBI of differential lipids in *H. congolense* WG10 planktonic cultures were higher under 20% salinity relative to 13%. These consolidate observations of this earlier work (6) that increased membrane fluidity is critical to high salinity tolerance in planktonic shale bacteria. However, in biofilm cells, we found that the MCL and DBI of differential lipids decreased by 4-fold under 20% salinity relative to the optimum. This indicates that under biofilm growth, which is typical in fractured shale reservoirs, *H. congolense* WG10’s adaptation to hypersalinity involves reducing the fluidity and thickness of its plasma membrane. There are no precedents for this observation, however, we believe this biophysical alteration might in some ways be necessary to promote more efficient intercellular interactions. Furthermore, in contrast to the aforementioned prior study (6) where DBI and MCL were reported to be significantly increased in *H. congolense* WG10 planktonic cells under the higher HRT vs the lower (at optimal salinity), the DBI and MCL of differential lipids in both *H. congolense* WG10 and the mixed consortia were barely influenced by HRT in the present investigation. In addition, we found that biofilm growth might induce an increase in membrane fluidity (estimated as DBI) and thickness (estimated as MCL) in *H. congolense* WG10. This contradicts other studies (7,8). It is important to keep in mind that the fatty acid-level resolution of LC-MS/MS analysis is often unreliable due to poor ionization and matrix effects (9). Therefore, these reported fatty acid trends require further investigations before broader scale deductions can be drawn from them.

**Supplementary figures legends**

**Figure S1.** Phospholipase D catalyzes the hydrolysis and transphosphatidylation of phosphatidylcholine (PC). XOH, alcohol.

**Figure S2.** Hierarchical clustering heat map of the 50 most discriminating lipid species (according to One-way ANOVA) collected in negative (A) and positive (B) ionization modes from *H. congolense* WG10 samples, produced fluid enrichment cultures and uncultivated field (filter) consortia. S7, S13 and S20 represent salinity gradients (%NaCl), while 19.2, 24, 48 and 72 are hydraulic retention times in hours (h). HC, *Halanaerobium congolense*; PEL, Planktonic cell pellet; BF, Biofilm; PFE, Produced fluid enrichment; CL, Cardiolipin; PG, Phosphatidylglycerol; DG, Diacylglycerol; PE, Phosphatidylethanolamine; PC, Phosphatidylcholine; PA, Phosphatidic acid; TG, Triacylglycerol; HexCer, Hexosylceramide; D19, December 2019; J20, July 2020; D20, December 2020; M21, May 2021.

**Figure S3**.  **Machine learning-based predictive modelling of *H. congolense* WG10 biofilm growth**. (A) Area under the curve (AUC) based on the cross-validation of the 15-feature Random forests (RF) model. (B) Predictive accuracies of RF models with different number of features.

**Figure S4.** Double bond index (DBI) and mean chain length (MCL) variations of differential (*P* < 0.04 & FC > 1.5) lipids in *H. congolense* and fluid enrichment cultures under different incubation conditions (salinity and HRT) and growth mode (biofilm vs. planktonic).

**Table S1.** Lipids undergoing significant change (*P* < 0.05 & |Log2Fold Change (FC)| > 1.5) in *H. congolense* WG10 planktonic cultures grown in chemostats (24 h HRT) under 13% vs. 20% NaCl.

| **Common Name** | **FC (20%/13%)** | ***P-*value** |
| --- | --- | --- |
| PE(16:0/18:1);PE(16:1/18:0) | 9.985932 | 0.000115 |
| PG(35:1) | 9.294973 | 0.000587 |
| PG(14:1/15:1) | 8.528971 | 0.016953 |
| PE(18:1/18:1) | 7.890022 | 0.035969 |
| PE(16:0/16:1);PE(14:0/18:1) | 7.780095 | 0.046979 |
| CL(16:0/18:1/18:1/18:1) | 5.460743 | 0.021868 |
| CL(14:0/16:1/16:1/16:1) | 3.936032 | 0.013857 |
| PG(12:0/12:0) | 3.746718 | 0.039057 |
| PE(16:0/17:1) | 3.047684 | 0.000702 |
| CL(16:0/16:1/16:0/17:1) | 2.887061 | 0.011485 |
| DG(14:1/16:1/0:0) | 2.726987 | 0.033868 |
| PG(18:0/0:0) | 2.506776 | 0.040333 |
| DG(16:0/18:1/0:0) | 2.376803 | 0.000176 |
| DG(14:0/16:1/0:0)_B;DG(14:1/16:0/0:0) | 1.958971 | 0.041139 |
| DG(16:1/0:0/16:1) | 1.882863 | 0.00731 |
| TG(40:0) | -1.51054 | 0.024065 |
| PE(19:1/0:0)_B | -7.68175 | 0.016535 |
| PE(17:1/0:0)_B | -10.0658 | 1.29E-06 |

PE, Phosphatidylethanolamine; PG, Phosphatidylglycerol; DG, Diacylglycerol; CL, Cardiolipin; TG, Triacylglycerol.

**Table S2.** Lipids undergoing significant change (*P* < 0.05 & |Log2Fold Change (FC)| > 1.5) in *H. congolense* WG10 biofilm cultures grown under 13% vs. 20% NaCl.

| **Common Name** | **FC (20%/13%)** | ***P-*value** |
| --- | --- | --- |
| PC(18:0/19:1) | -14.092 | 6.32E-05 |
| PC(16:0/17:1) | -5.55137 | 0.008161 |
| PC(16:0/19:1);PC(17:1/18:0) | -5.31517 | 0.004685 |
| PC(16:1/17:1) | -4.74339 | 0.04359 |
| PC(17:1/19:1) | -4.68119 | 0.006291 |
| PC(16:1/19:1);PC(17:1/18:1) | -3.74766 | 0.010408 |
| PC(18:1/19:1) | -3.55355 | 0.013147 |
| PG(18:0/18:0) | -3.33995 | 0.015335 |
| PC(16:0/16:1) | -2.8857 | 0.030707 |
| PC(16:0/18:1);PC(16:1/18:0) | -2.74156 | 0.031276 |
| PC(16:0/16:0) | -1.99707 | 0.029671 |
| PA(16:0/16:0) | -1.69732 | 0.003839 |
| PC(18:0/18:1) | -1.69014 | 0.048095 |
| PA(16:0/18:0) | -1.6241 | 0.005807 |
| PG(18:1/0:0)_B | 1.806945 | 0.015541 |
| PG(18:1/0:0)_A | 2.558337 | 0.03944 |

PA, Phosphatidic acid; PC, Phosphatidylcholine; PG, Phosphatidylglycerol.

**Table S3.** Lipids undergoing significant change (*P* < 0.05 & |Log2Fold Change (FC)| > 1.5) in *H. congolense* WG10 during biofilm growth vs. planktonic (48 h HRT), under 13% NaCl.

| **Common Name** | **FC (Biofilm/Planktonic)** | ***P-*value** |
| --- | --- | --- |
| PC(16:0/16:1) | 18.42342169 | 0.000273 |
| PE(19:1/0:0)_A | 18.01209289 | 0.001351 |
| PE(18:0/19:1) | 16.98429815 | 0.002287 |
| CL(18:1/18:1/18:1/19:1) | 16.91240858 | 0.00204 |
| PC(17:1/19:1) | 16.72403537 | 0.000404 |
| PC(16:1/19:1);PC(17:1/18:1) | 16.67097852 | 0.000376 |
| PC(19:1/19:1)_B | 16.59350161 | 0.000481 |
| PC(18:0/18:1) | 15.30350244 | 0.000387 |
| PC(18:0/19:1) | 14.09199164 | 0.000852 |
| PC(16:1/17:1) | 13.58505898 | 0.001668 |
| PC(0:0/18:1)_A | 13.33684605 | 0.001868 |
| PC(16:0/18:0) | 12.66362696 | 0.000598 |
| PE(17:1/18:0);PE(16:0/19:1) | 11.24072479 | 0.017156 |
| PE(19:1/19:1) | 11.24062466 | 0.012966 |
| PE(18:1/19:1) | 10.71945261 | 0.020683 |
| PE(16:0/17:1) | 10.34327623 | 0.024767 |
| PE(17:1/17:1) | 10.09700864 | 0.022477 |
| PE(17:1/18:1);PE(16:1/19:1) | 10.04849752 | 0.024214 |
| PE(16:1/17:1) | 9.818733134 | 0.016177 |
| PE(18:1/0:0)_A | 9.791153323 | 0.02043 |
| PE(18:0/18:1) | 9.603495759 | 0.023258 |
| PC(18:1/19:1) | 9.281919826 | 0.032837 |
| PE(16:0/16:1);PE(14:0/18:1) | 9.135669661 | 0.000655 |
| PE(14:0/16:0) | 8.808822348 | 0.02971 |
| PE(19:1/0:0)_B | 8.399134217 | 0.028468 |
| PG(19:1/19:1)_B | 8.328406281 | 0.049477 |
| PC(18:1/18:1) | 8.318063196 | 0.046197 |
| PE(16:0/16:0) | 8.174945925 | 0.042954 |
| CL(16:0/18:1/16:0/18:1) | 7.395362274 | 0.014093 |
| PG(17:1/19:1) | 6.768285591 | 0.04221 |
| PG(18:1/18:1) | 6.556494794 | 0.002224 |
| PG(16:0/18:1) | 4.074823076 | 0.007217 |
| TG(54:2) | 3.424205925 | 0.011527 |
| PG(16:0/18:0) | 3.279928114 | 0.002067 |
| CL(16:0/16:0/16:0/18:1) | 3.228540912 | 0.013344 |
| TG(48:1) | 3.192713796 | 0.029872 |
| PG(16:1/18:0)_A | 3.189260717 | 0.002914 |
| PG(16:1/18:1) | 3.136298873 | 0.001488 |
| PG(16:1/18:0)_B | 3.036358389 | 0.003521 |
| CL(14:0/16:1/16:1/16:1) | 2.94469069 | 0.019106 |
| TG(46:0) | 2.911468432 | 0.01021 |
| DG(16:1/0:0/16:1) | 2.869431645 | 0.020674 |
| PG(15:0/18:0)_B | 2.549482961 | 0.006025 |
| DG(32:1) | 2.543881601 | 0.006231 |
| PG(16:0/16:1)_A | 2.460681681 | 0.005964 |
| PG(16:0/17:1) | 2.44273359 | 0.000488 |
| PG(16:1/16:1) | 2.423811407 | 0.002903 |
| PG(16:0/16:0);PG(14:0/18:0) | 2.312310866 | 0.003383 |
| PG(14:1/16:0) | 2.302213793 | 0.001268 |
| PG(16:1/17:0) | 2.256917408 | 0.000361 |
| TG(44:0) | 2.196662746 | 0.000629 |
| TG(42:0) | 2.13039882 | 0.006757 |
| PG(14:0/14:1)_B | 2.069614518 | 0.001921 |
| DG(12:0/14:0/0:0) | 2.06569889 | 0.002 |
| PG(14:0/16:1) | 2.038327094 | 0.00098 |
| PG(14:0/16:0) | 2.021730383 | 0.003215 |
| PG(P-16:0/16:1) | 2.000990448 | 0.002033 |
| TG(52:0) | 1.883996046 | 0.014219 |
| PG(16:0/16:1)_B | 1.882338545 | 0.002749 |
| TG(40:0) | 1.859648062 | 0.000165 |
| PG(13:0/16:1) | 1.844809034 | 0.019715 |
| CL(16:0/16:1/16:0/17:1) | 1.841547851 | 6.55E-05 |
| PG(15:0/16:1);PG(15:1/16:0) | 1.810739061 | 2.58E-05 |
| PG(18:1/0:0)_B | 1.773621823 | 0.009918 |
| CL(16:0/17:1/18:1/16:1) | 1.726225513 | 0.032292 |
| DG(34:2) | 1.724565115 | 0.017515 |
| PG(14:0/14:0) | 1.692752474 | 0.000886 |
| PG(P-16:0/14:0) | 1.666995498 | 0.003199 |
| CL(17:1/16:0/16:1/17:1) | 1.665618667 | 0.015764 |
| PG(31:1) | 1.664820699 | 0.003572 |
| PG(16:1/0:0) | 1.640891587 | 0.002218 |
| PG(15:1/17:1) | 1.639487073 | 0.001274 |
| PG(15:0/16:0) | 1.613854867 | 0.002863 |
| PG(P-16:0/16:0) | 1.541934289 | 0.004631 |
| PG(12:0/13:0) | -2.315767982 | 0.000109 |
| HexCer(d18:2/16:1)_B | -8.488442476 | 0.048769 |

PC, Phosphatidylcholine; PE, Phosphatidylethanolamine; PG, Phosphatidylglycerol; DG, Diacylglycerol; CL, Cardiolipin; TG, Triacylglycerol; HexCer, Hexosylceramide.

**Table S4.** Lipids undergoing significant change (*P* < 0.05 & |Log2Fold Change (FC)| > 1.5) in mixed microbial consortia in produced fluids enriched as biofilms vs. planktons (48 h HRT).

| **Common Name** | **FC (Biofilm/Planktonic)** | ***P-*value** |
| --- | --- | --- |
| PC(16:0/18:0) | 1.814956 | 0.017518 |
| PC(18:0/19:1) | 1.578926 | 0.019086 |

PC, Phosphatidylcholine

**Table S5.** Lipids undergoing significant change (*P* < 0.05 & |Log2Fold Change (FC)| > 1.5) in *H. congolense* WG10 planktonic cultures grown at 13% NaCl under 19.2 h (lowest) vs 48 h (highest) HRT.

| **Common Name** | **FC (48 h/19.2 h)** | ***P-*value** |
| --- | --- | --- |
| PG(19:1/0:0)_B | 9.887434 | 0.049853 |
| PG(12:0/13:0) | 3.935999 | 0.004234 |
| PG(18:1/18:1) | 3.387707 | 0.005895 |
| PG(12:0/0:0) | 2.755364 | 0.01389 |
| PG(16:1/20:0);PG(18:0/18:1) | 2.169967 | 0.025216 |
| PG(13:0/14:0) | 2.111622 | 0.015093 |
| PG(14:1/14:1) | -1.86509 | 0.017244 |
| PE(17:1/0:0)_B | -2.02045 | 0.026899 |
| CL(17:1/16:1/16:1/16:1) | -2.34835 | 0.009317 |
| DG(15:1/16:1/0:0) | -2.55928 | 0.039806 |
| CL(14:0/16:1/16:1/16:1) | -2.60089 | 0.001994 |
| DG(14:1/16:1/0:0) | -3.39663 | 0.016273 |
| CL(14:1/16:1/16:1/16:1) | -3.83974 | 0.001797 |

CL, Cardiolipin; PE, Phosphatidylethanolamine; PG, Phosphatidylglycerol; DG, Diacylglycerol

**Table S6.** Lipids undergoing significant change (*P* < 0.05 & |Log2Fold Change (FC)| > 1.5) in produced fluid enrichment planktonic cultures enriched under 19.2 h (lowest) vs 72 h (highest) HRT.

| **Common Name** | **FC (72 h/19.2 h)** | ***P-*value** |
| --- | --- | --- |
| CL(14:1/16:1/16:1/16:1) | 12.86029 | 4.61E-07 |
| CL(17:1/16:1/16:1/16:1) | 12.80119 | 9.57E-06 |
| PA(16:0/16:0) | 12.61375 | 2.03E-05 |
| PG(P-16:0/16:1) | 12.54334 | 1.51E-05 |
| PG(P-16:0/14:0) | 11.41802 | 8.82E-06 |
| DG(15:0/16:0/0:0);DG(14:0/17:0/0:0) | 11.09119 | 1.05E-05 |
| PA(16:0/18:0) | 11.03607 | 7.68E-07 |
| CL(14:0/16:1/16:1/16:1) | 7.058822 | 0.017452 |
| TG(38:0) | 5.91441 | 3.80E-05 |
| DG(16:1/0:0/16:1) | 5.835831 | 2.76E-05 |
| DG(14:0/16:1/0:0)_A | 5.504021 | 0.00021 |
| PG(14:0/14:1)_B | 5.000328 | 0.028177 |
| DG(14:0/16:1/0:0)_B;DG(14:1/16:0/0:0) | 4.587794 | 0.000249 |
| PG(15:1/17:1) | 4.552592 | 0.000356 |
| DG(14:1/16:1/0:0) | 4.293553 | 9.54E-05 |
| PG(14:1/14:1) | 3.832545 | 0.035211 |
| PG(14:1/16:1) | 3.815439 | 0.000101 |
| PG(14:0/14:1)_A;PG(12:0/16:1) | 3.644003 | 0.000161 |
| DG(16:1/17:1/0:0) | 3.233923 | 0.000409 |
| DG(12:0/14:0/0:0) | 3.233742 | 0.000235 |
| PG(15:1/16:1) | 2.954893 | 0.000252 |
| PG(12:0/14:0) | 2.809179 | 0.000478 |
| PG(14:0/16:1) | 2.716755 | 0.000207 |
| PG(14:0/14:0) | 2.358268 | 0.001125 |
| DG(12:0/14:1/0:0) | 2.16797 | 0.001925 |
| PG(14:0/0:0)_B | 2.092044 | 0.02356 |
| PG(14:0/15:1) | 2.058085 | 0.00105 |
| PG(13:0/16:0);PG(14:0/15:0) | 2.031547 | 0.001295 |
| DG(32:1) | 1.906149 | 0.003748 |
| PG(12:0/14:1) | 1.612325 | 0.00612 |
| PG(14:0/0:0)_A | 1.597344 | 0.005982 |
| PE(16:1/16:1) | -1.52859 | 0.023713 |
| TG(54:3) | -1.58525 | 0.000877 |
| DG(16:0/18:1/0:0) | -1.75487 | 0.002409 |
| TG(52:2) | -1.76915 | 0.002157 |
| TG(54:2) | -1.92053 | 0.000273 |
| PE(16:1/18:1)_A | -1.93121 | 0.010015 |
| PC(16:0/18:1);PC(16:1/18:0) | -2.19467 | 0.0031 |
| PG(16:0/16:1)_B | -2.2264 | 0.004109 |
| TG(52:1) | -2.23415 | 0.000263 |
| PE(18:0/18:1) | -2.2434 | 0.047204 |
| PC(18:0/18:1) | -2.34545 | 0.002808 |
| PE(18:0/0:0) | -2.3545 | 0.036594 |
| PG(17:1/17:1) | -2.43261 | 0.002494 |
| PE(16:0/18:1);PE(16:1/18:0) | -2.48567 | 0.014104 |
| PG(16:0/16:1)_A | -2.60859 | 0.000885 |
| PC(16:0/16:1) | -2.6362 | 0.0012 |
| PC(0:0/18:0) | -2.87557 | 0.007227 |
| PG(16:1/18:1) | -2.93628 | 0.009161 |
| PC(16:1/17:1) | -3.01162 | 0.00495 |
| PG(15:0/16:0) | -3.23036 | 0.002064 |
| PG(16:1/20:0);PG(18:0/18:1) | -3.28407 | 0.015354 |
| PC(18:1/19:1) | -3.37588 | 0.00051 |
| PE(16:0/18:0) | -3.43497 | 0.000679 |
| PC(16:1/19:1);PC(17:1/18:1) | -3.54991 | 0.000217 |
| PC(19:1/19:1)_B | -3.63705 | 0.000539 |
| PE(16:1/17:1) | -3.67164 | 0.001436 |
| PC(17:1/19:1) | -3.73867 | 0.000282 |
| PE(17:1/18:1);PE(16:1/19:1) | -3.73977 | 0.010968 |
| PE(16:0/16:1);PE(14:0/18:1) | -3.74068 | 0.000699 |
| TG(36:0) | -3.80205 | 0.000425 |
| PC(18:0/19:1) | -3.84204 | 0.000528 |
| PG(16:0/18:1) | -3.84526 | 0.00426 |
| PG(16:1/18:0)_A | -3.84526 | 0.00426 |
| PC(17:1/0:0)_A | -3.84602 | 0.020969 |
| PE(17:1/17:1) | -3.90131 | 0.000229 |
| PC(16:0/19:1);PC(17:1/18:0) | -3.98569 | 0.000437 |
| PE(16:0/16:0) | -4.02471 | 6.91E-06 |
| PE(19:1/19:1) | -4.03118 | 0.000127 |
| PE(18:0/19:1) | -4.04209 | 6.66E-05 |
| PE(14:0/16:0) | -4.0554 | 0.000156 |
| PE(17:1/19:1) | -4.07303 | 0.000538 |
| PE(17:1/18:0);PE(16:0/19:1) | -4.21496 | 3.31E-05 |
| PG(16:0/17:1) | -4.31 | 0.000153 |
| PG(16:1/19:1);PG(17:1/18:1) | -4.42791 | 0.002649 |
| PC(16:0/16:0) | -4.55425 | 0.000983 |
| PC(16:0/18:0) | -4.61853 | 0.000504 |
| PE(18:1/19:1) | -4.68262 | 0.000533 |
| PC(19:1/19:1)_A | -4.68782 | 0.000287 |
| PC(16:0/17:1) | -4.69421 | 0.000135 |
| PE(14:0/16:1) | -4.75423 | 0.034709 |
| PG(18:1/19:1)_A | -4.9211 | 0.003448 |
| PG(16:0/16:0);PG(14:0/18:0) | -4.99939 | 0.000212 |
| PG(16:1/17:0) | -5.01526 | 0.002885 |
| PG(35:1) | -5.15768 | 8.75E-05 |
| PE(16:0/17:1) | -5.17155 | 0.000149 |
| PG(18:0/19:1) | -5.32651 | 0.000264 |
| PG(17:1/19:1) | -5.4218 | 5.53E-05 |
| PG(19:1/19:1)_B | -5.64072 | 0.000134 |
| PG(16:0/18:0) | -5.78536 | 0.000154 |
| PG(18:1/19:1)_B | -6.12331 | 0.036061 |
| PG(15:0/18:0)_B | -6.73398 | 0.019061 |
| CL(16:0/16:0/16:0/18:1) | -11.3123 | 0.000398 |
| PG(15:0/18:0)_A;PG(16:0/17:0) | -11.4381 | 0.000298 |
| CL(16:0/18:1/16:0/18:1) | -12.2432 | 0.000151 |
| PG(18:0/18:0) | -12.8973 | 7.15E-05 |
| PE(19:1/0:0)_A | -13.1918 | 8.08E-05 |
| CL(16:0/17:1/16:0/18:1) | -13.2539 | 0.000376 |
| PC(0:0/16:0) | -13.9134 | 2.62E-05 |
| PG(19:1/19:1)_A | -14.2288 | 2.25E-05 |

PC, Phosphatidylcholine; PE, Phosphatidylethanolamine; PG, Phosphatidylglycerol; DG, Diacylglycerol; CL, Cardiolipin; TG, Triacylglycerol

**REFERENCES**

1. Yang SF, Freer S, Benson AA. Transphosphatidylation by Phospholipase D. J Biol Chem. 1967 Feb 10;242(3):477–84.

2. Campomanes P, Zoni V, Vanni S. Local accumulation of diacylglycerol alters membrane properties nonlinearly due to its transbilayer activity. Commun Chem. 2019 Jun 25;2(1):1–8.

3. Parsons JB, Rock CO. Bacterial Lipids: Metabolism and Membrane Homeostasis. Prog Lipid Res. 2013 Jul;52(3):249–76.

4. Van Horn WD, Sanders CR. Prokaryotic Diacylglycerol Kinase and Undecaprenol Kinase. Annu Rev Biophys. 2012;41:81–101.

5. Wältermann M, Stöveken T, Steinbüchel A. Key enzymes for biosynthesis of neutral lipid storage compounds in prokaryotes: Properties, function and occurrence of wax ester synthases/acyl-CoA:diacylglycerol acyltransferases. Biochimie. 2007 Feb 1;89(2):230–42.

6. Ugwuodo CJ, Colosimo F, Adhikari J, Shen Y, Badireddy AR, Mouser PJ. Salinity and hydraulic retention time induce membrane phospholipid acyl chain remodeling in Halanaerobium congolense WG10 and mixed cultures from hydraulically fractured shale wells. Front Microbiol. 2022;13:1023575.

7. Dubois-Brissonnet F, Trotier E, Briandet R. The Biofilm Lifestyle Involves an Increase in Bacterial Membrane Saturated Fatty Acids. Front Microbiol [Internet]. 2016 [cited 2022 Jun 26];7. Available from: https://www.frontiersin.org/article/10.3389/fmicb.2016.01673

8. Chao J, Wolfaardt GM, Arts MT. Characterization of Pseudomonas aeruginosa fatty acid profiles in biofilms and batch planktonic cultures. Can J Microbiol. 2010 Dec;56(12):1028–39.

9. Feng X, Wang J, Tang Z, Chen B, Hou X, Li J, et al. A strategy for accurately and sensitively quantifying free and esterified fatty acids using liquid chromatography mass spectrometry. Front Nutr. 2022 Aug 3;9:977076.
